# Fracture Toughness of Veneering Ceramics for Fused to Metal (PFM) and Zirconia Dental Restorative Materials

**DOI:** 10.6028/jres.115.024

**Published:** 2010-10-01

**Authors:** Janet B. Quinn, George D. Quinn, Veeraraghaven Sundar

**Affiliations:** American Dental Association Foundation, Paffenberger Research Center, National Institute of Standards and Technology, Gaithersburg, MD; Dentsply Prosthetics, 550 West College Avenue, York, PA

**Keywords:** dental restorations, fracture toughness, hardness, PFM, surface crack in flexure, veneering ceramic, zirconia

## Abstract

Veneering ceramics designed to be used with modern zirconia framework restorations have been reported to fracture occasionally in vivo. The fracture toughness of such veneering ceramics was measured and compared to that of conventional feldspathic porcelain veneering ceramics for metal framework restorations. The fracture toughness of the leucite free veneer was measured to be 0.73 MPa m ± 0.02 MPa m, which is less than that for the porcelain fused to metal (PFM) veneering ceramic: 1.10 MPa ± 0.2 MPa. (Uncertainties are one standard deviation unless otherwise noted.) The surface crack in flexure (SCF) method was suitable for both materials, but precrack identification was difficult for the leucite containing feldspathic porcelain PFM veneer.

## 1. Introduction

Over the last 15 years, zirconia has seen use as a substructure material for the fabrication of fixed prosthodontic restorations, i.e., crowns and bridges. Zirconia is regarded as offering superior strength, toughness and reliability over other ceramic materials due to the transformation toughening mechanisms of its microstructure [1–3]. Clinical studies are now available that support zirconia’s performance potential, with indications of expanded functionality compared with other ceramics, such as use in long span bridges [4–8]. The primary issues noted in such studies were not related to framework integrity, but rather chipping, wear, and fracture of the veneering ceramic [9, 10]. A recent study suggests that such chipping could also be an issue around endodontic access openings for all-ceramic crowns [11].

These observations prompted thinking about differences between porcelain fused to metal (PFM) veneering ceramics, which have been used for over 40 years [12], and the more recently developed veneering ceramics intended for zirconia. The veneers exhibit some compositional and microstructural differences, but are manufactured to identical international standards in terms of mechanical properties [13]. Veneer compositions are adjusted so that their thermal expansions are optimized for the framework materials they are designed to be used with. Hence, PFM veneers are different than those for zirconia. Based on clinical observations, as well as the in-vitro materials data, the question arises whether zirconia veneering ceramics are more susceptible to chipping than PFM veneering ceramics.

In a companion paper, the fracture resistance of the respective veneers were measured by their edge chip resistance [14]. Bilayer PFM and PFZ (porcelain fused to zirconia) test coupons were subjected to edge loading by a sharp conical indenter near the edge. Resistance to fracture was assessed by measuring the force necessary to flake a chip off the side of the test coupon. Surprisingly, there was very little difference in edge chip resistance between the PFM and PFZ veneers.

**Fig. 1:**
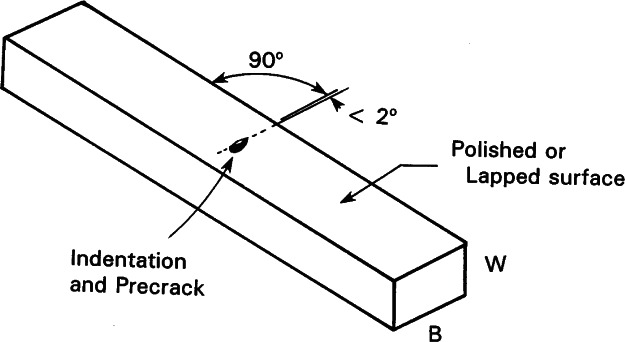
The surface crack in flexure (SCF) method. An aligned Knoop indentation is used to create a semielliptical precrack on a bend bar.

**Fig. 2:**
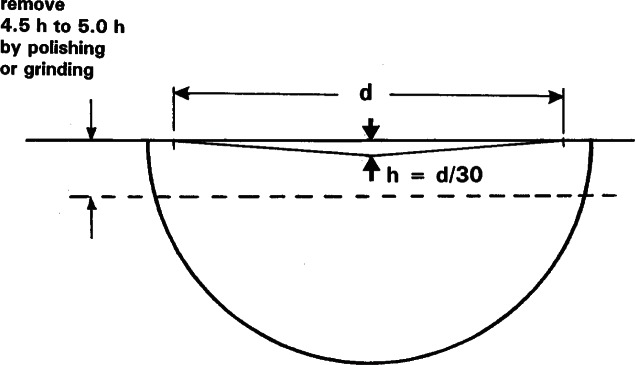
Schematic of the indentation that is d long and h deep and the precrack that pops in underneath it. Between 4.5 h and 5.0 h must be removed to eliminate the indentation damage zone and its associated residual stresses.

**Fig. 3:**
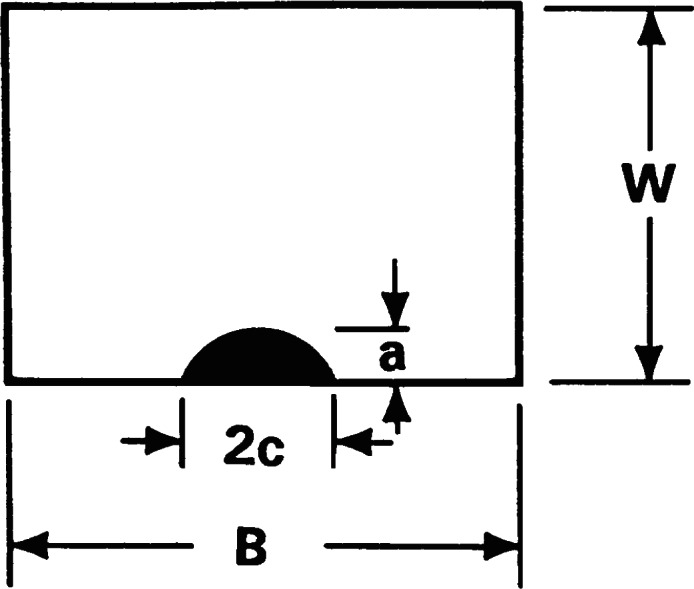
Schematic of the beam cross section with a semielliptical surface crack. The precrack size is exaggerated in this view for clarity.

To further examine potential differences in the fracture resistance of the respective veneers, the fracture toughness, K_Ic_, of each type of veneer was measured using the surface crack in flexure (SCF) method. In this method, which is schematically illustrated in Figs. 1–3, a Knoop indentation is positioned in the center of each flexure test bar at sufficient load to cause cracks to form. Crack orientation may be controlled by orienting the Knoop indentation axis. After indentation, 4.5 to 5 times the depth of the indent is removed from the surface of each bar. This is an important step, for it eliminates the residual stresses resulting from the indentation, and leaves a stress-free semi-elliptical crack in the bar surface. The bars are then tested in flexure and break at the semi-elliptical crack if it is large enough. The size of the crack is measured on the fracture surface of each specimen, and substituted into formulae for calculating K_Ic_, along with the specimen size and break stress. One advantage of the SCF method is that it gives a fracture toughness result germane to small cracks, of the order of size of the naturally occurring flaws in a brittle material. This method has been used previously for dental restoration ceramics [15] and has been standardized by ASTM Int. [16, 17], the International Organization for Standardization (ISO) [18], and the European Committee for Standards (CEN) [19]. It was the focus of a major Versailles Advanced Materials and Standards (VAMAS) international round robin [20–22]. It also was one of the core methods used to prepare Standard Reference Material (SRM) 2100, the world’s only reference material for the property fracture toughness, K_Ic_, [23, 24]. SCF results were identical to within 0.01 MPa√m to those from chevron notch in bending and single-edge precracked beam experiments. In other words, the three methods produced virtually identical results on the reference material, which has a very fine grain size and had a flat R-curve (such that fracture toughness was independent of the crack size). Although the method has been widely used to measure fracture toughness of a wide range of ceramics (e.g., [25]), it does have limitations. The SCF method will not work on all ceramic materials. The following criteria must be met:

1. The material must be hard and brittle.
2. It must be possible to detect the precracks on the fracture surface after fracture.
3. The precrack size should be larger than the natural flaws in the material.

Difficulties arise if the material is coarse-grained, porous, too tough, or too soft. It is difficult to detect the precracks if the material is coarse grained or porous. Also, if the material is porous or soft, cracks will not form under the indentation. If the material has too great a fracture toughness, then only small precracks form and they may be removed when the indentation damage zone is removed in the polishing step.

The goals of this work were to ascertain whether the SCF method was suitable for dental veneering ceramics and whether a conventional PFM veneer had a different resistance to fracture than a new PFZ veneer.

## 2. Materials and Methods

### 2.1 Materials

A vacuum dental furnace (model: Centurion VPC, Dentsply Prosthetics, York, PA) was used to fire six 2.8 mm × 3.6 mm × 42 mm long beam specimens in rectangular molds. The PFM veneer was Ceramco 3 (Dentsply Prosthetics, York, PA)^1^, a feldspathic veneering porcelain containing about 0.30 volume fraction leucite. The veneer for the zirconia, Ceramco PFZ (Dentsply Prosthetics, York, PA), was designed to be used with a 3 mole % yttria stabilized zirconia (Cercon) made by the same manufacturer. This veneer is a feldspathic porcelain with negligible leucite. The Ceramco 3 veneer for the alloy had a heat up rate of 55°C/min to a peak firing temperature of 960°C and 0 min hold. The Ceramco PFZ veneer for the zirconia had a heat up rate of 60°C/min to a peak temperature of 900°C and a short 15 s hold on the first firing and 890°C and 0 min hold on the second firing. The specimens were rough-polished on the four long surfaces to eliminate any bowing or small surface lumps. The two 3.6 mm wide surfaces were more carefully polished with successively smaller grits down to 1200 grit. A companion paper has additional information including edge chip resistance and Knoop hardness measurements for both veneering ceramics [14].

### 2.2 Methods: SCF Fracture Toughness

Knoop indentations with a load of 39.2 N (4 kg mass on the indenter) were placed on one of the wide 3.6 mm surfaces. The indentation and the residual stress-creating damage zone associated with the indentation were removed to a depth of 4.5 to 5.0 times the depth of the indentation, h, by hand grinding with 180 grit silicon carbide abrasive papers. The Knoop indentation lengths, d, were of the order of 370 μm for the zirconia veneer so that the indentation depths (≈ 1/30th of the diagonal length) were about 12 μm deep. Thus, about 60 μm of material was removed by the hand grinding. Lateral cracks around the indentation were detected for the PFZ veneer, but they were removed by the grinding. The indentations were about 440 μm long for the PFM veneer, so about 73 μm was removed by hand grinding for each bar.

Fractographic techniques are used to detect and measure the precrack on the fracture surface after test specimen fracture [26]. The precrack size was measured for each test specimen. The precracks were very easy to detect on the zirconia veneer. A stereoptical microscope (Leica MZ16, Wetzlar, Germany) with a traversing stage with a resolution of 1 μm was used to measure the precrack width, 2c, and depth, a, on the fracture surfaces. The precacks were far more difficult to measure in the PFM veneer since the fracture surface was much rougher. One half of each specimen was coated with a thin sputter applied gold coating (such as is done for scanning electron microscope examination) in order to facilitate viewing with the stereoptical microscope. The coating cut down on internal light scattering in this translucent material and improved contrast.

Bars were broken in three-point flexure with a 20 mm span, taking care that the precrack was well centered under the middle loading roller. The 42 mm long bars could have been tested in four-point loading with 20 mm and 40 mm fixture spans, but the shorter span fixture was used so that two breaks could be obtained with some of the test pieces. Four-point loading is usually recommended for the SCF method [16–21, 23–25], to ensure that the precrack is within a constant stress region, obviating the need for meticulous alignment in three-point testing. All testing was done in laboratory ambient conditions at a crosshead rate of 0.5 mm/min.

Fracture toughness (K_Ic_) was calculated from the formula for a semicircular or semielliptical surface crack in tension or flexure:

(1) $$K_{\text{Ic}}=Y\;\sigma \;\surd a$$

where Y is the stress intensity shape factor, σ is the flexure strength of the specimen (MPa), and a is the crack depth (m). Y is dimensionless and is a function of the crack size and shape and was individually calculated for each precrack. The solutions by Newman and Raju [27] were used. The maximum Y value from around the crack front periphery was used to compute K_Ic_. For the precracks in this study, this was often where the precrack intersected the tensile surface, but usually the Y values varied by less than 10 % around the precrack periphery. One surprise about the SCF method is that computed fracture toughness values are not especially sensitive to the measurement of the pre-crack size. This is due in part to the square root dependence of K_Ic_ on the crack size, but also due to an offsetting influence of Y on the crack size measurement. For example, as discussed in Scherrer et al., [15], multiple observers using different photos and measurements obtained K_Ic_ values that agreed on average to within 0.01 MPa√m for a dental feldspathic porcelain. Additional details about Y and the SCF method may be found in [15–21, 25].

## 3. Results

**Fig. 4:**
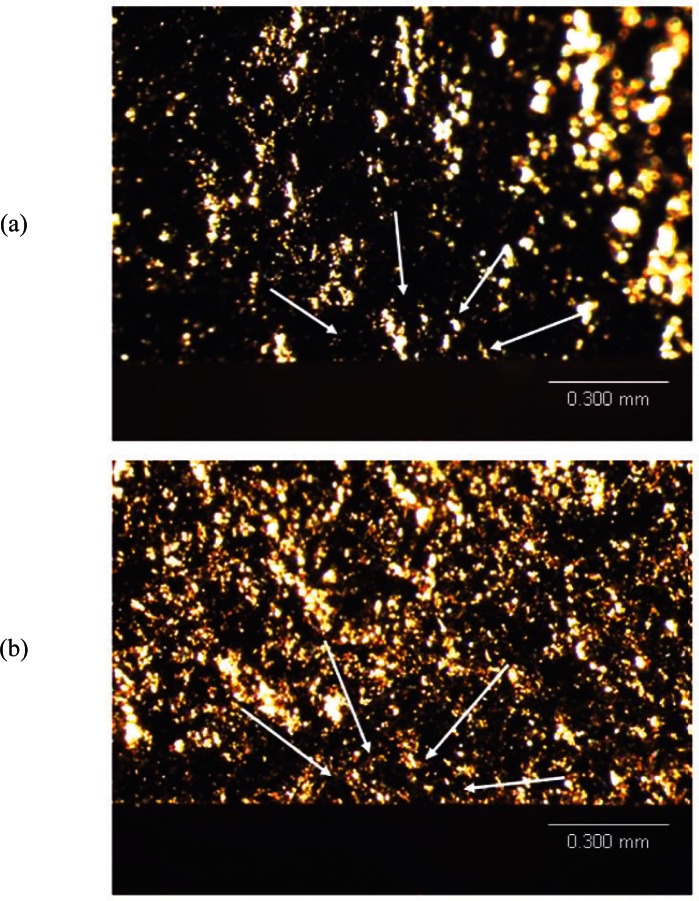

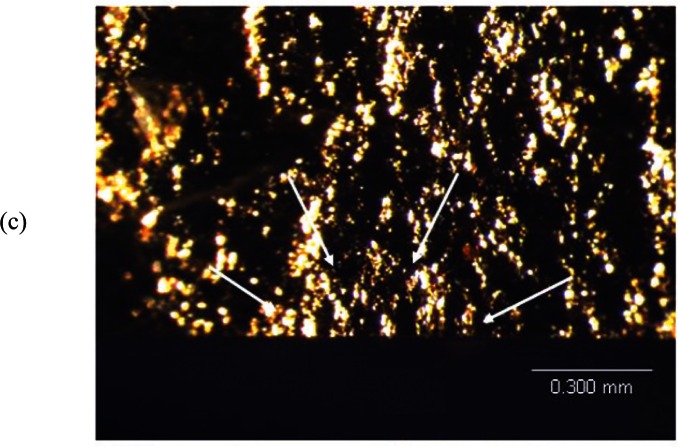
Examples of Precracks in the veneer for the PFM system (a), (b), and (c).

Precracks were very difficult to measure in the veneering ceramic for the PFM. Figure 4 shows some examples. The precracks were interpreted both from the photos and from direct viewing in the stereoptical microscope which gives a much clearer three-dimensional view. Our difficulty in interpreting pre-crack sizes with this material was similar to our previous experiences with another feldspathic porcelain [15]. In the latter study, scanning electron microscope examination was a very helpful adjunct to the optical examinations, but it was not necessary in this study. Only five of the six specimens produced measureable precracks. The precracks had depths that ranged from 141 μm to 226 μm, with an average of 162 μm. The precrack widths ranged from 339 μm to 534 μm with an average of 417 μm. Flexural strengths for the beam ranged from 61 MPa to 77 MPa. The fracture toughness was 1.10 MPa√m ± 0.13 MPa√m. (Uncertainties are one standard deviation unless otherwise noted.)

**Fig. 5:**
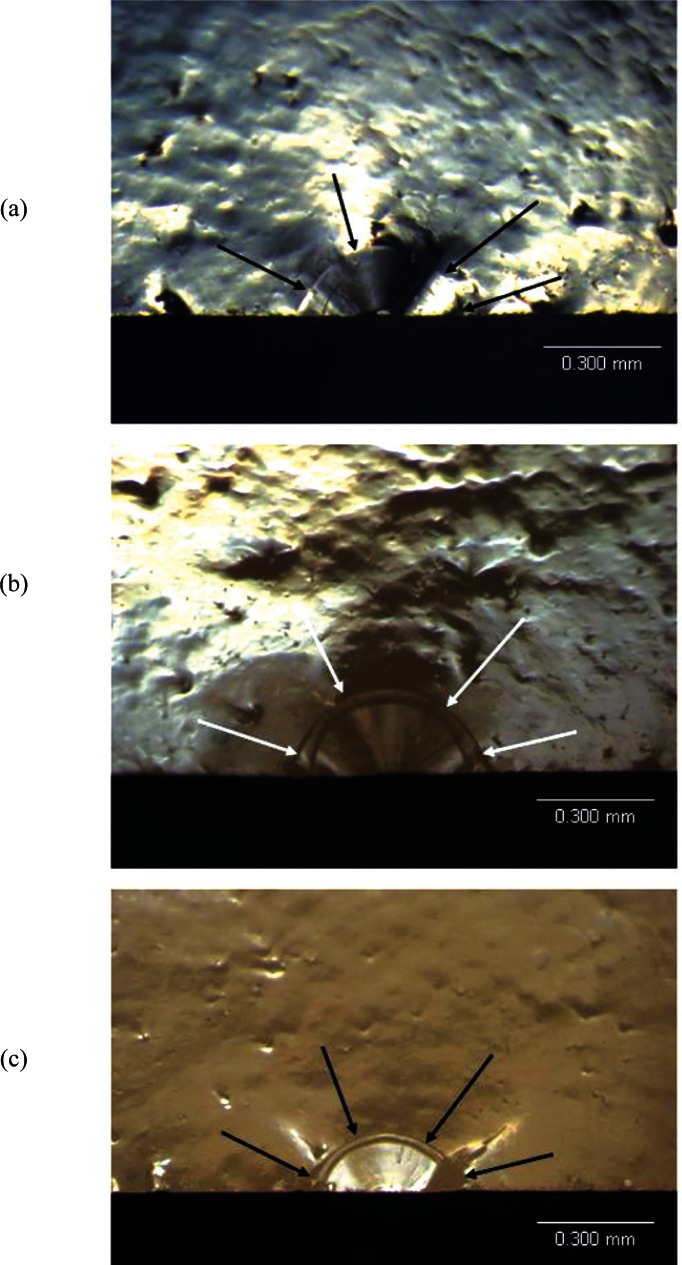

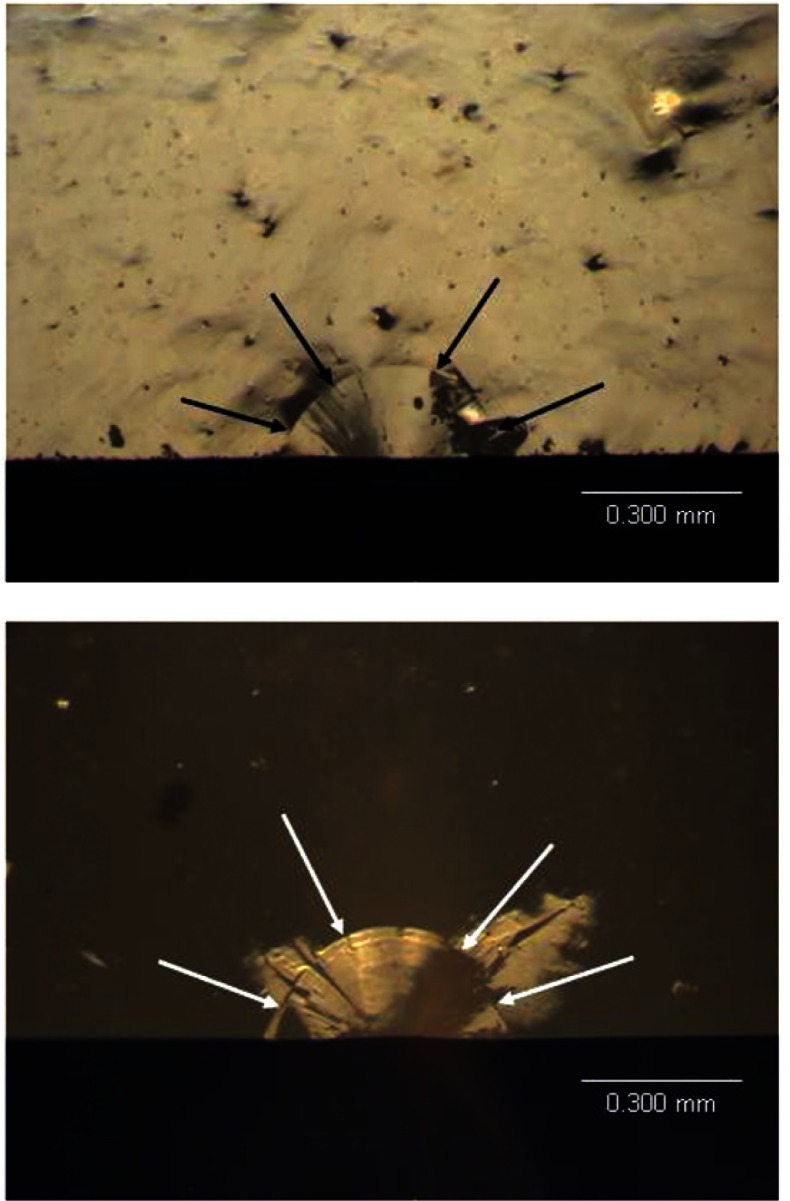
Precracks in the veneer for the PFZ system (a), (b), (c), and ((d) and (e).

In contrast, the semi-elliptical cracks in the Ceramco PFZ were very easy to measure. From the six specimens, five good breaks from the precracks were obtained as shown in Fig. 5, which shows the range of sizes and appearances. A large bubble caused fracture in another trial, and grinding scratches caused breakage in two other tests. The dark-light variations are optical effects from variable reflections of the precrack and the surrounding material. Several precracks showed concentric bands, suggesting that the precrack popped-in in multiple steps or that the precrack grew stably during the final fracture test. The largest outer crack was used for the fracture toughness calculations. The precracks ranged in size from depths of 157 μm to 221 μm, with an average of 192 μm. The precrack widths ranged from 403 μm to 505 μm with an average of 443 μm. Flexural strengths ranged from 39 MPa to 45 MPa. The average fracture toughness was 0.73 MPa√m with a standard deviation of only 0.02 MPa√m.

## 4. Discussion

The fracture toughness of the PFM veneer (1.10 MPa√m ± 0.13 MPa√m) is statistically significantly greater (50 %) than that for the PFZ veneer (0.73 MPa√m ± 0.02 MPa√m, P < 0.01 %, Students’ t distribution). This is not surprising since the PFM veneer that has leucite reinforcing crystals that improve fracture toughness. The leucite is also used to create thermal expansion compatibility with metal substructures. Our fracture toughness results are comparable to those previously measured by us using the single-edged V-notched beam method (0.99 MPa√m ± 0.06 MPa√m to 1.26 MPa√m ± 0.04 MPa√m) for similar feldspathic PFM porcelains [28]. Our result is also comparable to SCF results by Scherrer et al., [15]. In the latter study, specimens tested in an inert environment (dry nitrogen gas) had a fracture toughness of 1.02 MPa√m ± 0.01 MPa√m which was 0.09 MPa√m greater on average than results for experiments done under lab ambient conditions (0.93 MPa√m ± 0.06 MPa√m). The reduced fracture toughness was due to slow crack growth from water vapor in the air.

Leucite is not needed for thermal expansion purposes in the PFZ veneering ceramic since the thermal expansion of zirconia is much less than of dental noble metals. The PFZ veneer is a multiphase mixture of glass compositions. Accordingly, the fracture surfaces of the PFZ veneer were very flat and glassy in appearance. Indeed, the fracture toughness is comparable to but slightly less than values for common soda lime glasses (0.75 MPa√m to 0.80 MPa√m), possibly since slow crack growth may have occurred in our experiments that were conducted in laboratory ambient conditions.

These results are in contrast with previous findings [14] that showed the fracture resistance of both veneers were very similar. The earlier study used the edge chipping procedure to measure the resistance to chip fracture with a sharp indenter under load near a well defined edge of a test piece. The load (F_c_) necessary to cause a chip at a given distance (d_e_) from the edge varied with the square of the distance in accordance with:

(2) $$F_{c}=A\;d_{e}{}^{2}$$

where A was a proportionality constant. A was 407 N/mm^2^ for the zirconia veneer and 440 N/mm^2^ for the PFM veneer, an 8 % difference. Although some studies have shown that edge toughness scales with fracture toughness, our new results suggest fracture toughness and edge toughness are two different indices of resistance to fracture.

The SCF fracture toughness technique was successful in these instances, but interpretation of the precracks was very difficult in the case of the veneer for the PFM system. A simple dye penetration procedure to highlight the precracks would be a welcome step to facilitate interpretation, but past efforts have had mixed successes. Dyes are effective in some materials, but ineffective in others due to variations in wetabilty and the tightness of the rather small Knoop precracks. Although we were able to obtain valid fractures in three-point loading, four-point is much preferred and should be used in the future due to the strong stress gradients in the former. Future testing of such oxide ceramics should be done in inert environmental conditions to minimize possible interferences from slow crack growth.

The reduced fracture toughness of the veneering ceramic for the zirconia system could be an important factor in the difference in the clinical behavior. It has also been reported recently that residual stresses in the zirconia veneering ceramic may also contribute to their increased propensity to fracture [29, 30].

## 5. Conclusion

The fracture toughness of the leucite containing feldspathic porcelain veneering ceramic intended for use with PFMs was 1.10 MPa√m ± 0.13 MPa√m. It is greater than that for a veneering ceramic designed for zirconia: 0.73 MPa√mm ± 0.02 MPa√m. The SCF method was suitable for both materials, but precrack identification was difficult for the leucite containing feldspathic porcelain PFM veneering ceramic.

## Footnotes

1 Commercial products and equipment are identified only to specify adequately experimental procedures and does not imply endorsement by the authors, institutions or organizations supporting this work, nor does it imply that they are necessarily the best for the purpose.
